# Wheat Bran Phenolic Acids: Bioavailability and Stability in Whole Wheat-Based Foods

**DOI:** 10.3390/molecules200915666

**Published:** 2015-08-28

**Authors:** Barbara Laddomada, Sofia Caretto, Giovanni Mita

**Affiliations:** Istituto di Scienze delle Produzioni Alimentari, CNR, Via Prov.le Monteroni, 73100 Lecce, Italy; E-Mails: sofia.caretto@ispa.cnr.it (S.C.); giovanni.mita@ispa.cnr.it (G.M.)

**Keywords:** whole-wheat flour, phenolic acids, bioactive compounds, antioxidant activity, bio-accessibility, functional foods

## Abstract

Wheat bran is generally considered a byproduct of the flour milling industry, but it is a great source of fibers, minerals, and antioxidants that are important for human health. Phenolic acids are a specific class of wheat bran components that may act as antioxidants to prevent heart disease and to lower the incidence of colon cancer. Moreover, phenolic acids have anti-inflammatory properties that are potentially significant for the promotion of gastrointestinal health. Evidence on the beneficial effects of phenolic acids as well as of other wheat bran components is encouraging the use of wheat bran as an ingredient of functional foods. After an overview of the chemistry, function, and bioavailability of wheat phenolic acids, the discussion will focus on how technologies can allow the formulation of new, functional whole wheat products with enhanced health-promoting value and safety without renouncing the good-tasting standards that are required by consumers. Finally, this review summarizes the latest studies about the stability of phenolic acids in wheat foods fortified by the addition of wheat bran, pearled fractions, or wheat bran extracts.

## 1. Introduction

A number of epidemiological studies have demonstrated a dose-response relation between whole grain intake and the risk of chronic diseases, indicating a positive correlation between the amount of whole grain consumed and the associated health benefits [1,2,3]. Consumption of 2–3 servings per day (~48 g) of whole grains may reduce the risk of cardiovascular disease, cancer, and type 2 diabetes mellitus [4,5,6]. Whole grains have greater nutritional value than refined grains, which is due to the unique health-promoting components that exist in the bran and germ portions [3,7,8]. Particularly, wheat bran is rich in phenolic acids, which are mainly covalently cross-linked with cell wall polymers [9].

In view of the fact that the milling process commonly leads to isolating and employing only the starchy endosperm, the bran and germ portions are commonly discarded and destined for animal feeding. However, due to the up-and-coming findings on the beneficial effects of its components, the use of bran as a food ingredient is increasing. The main challenge for the addition of bran or widdlings in bakery and pasta processes is to overcome the adverse effects that they have on the quality of the final products [10,11,12]. Several strategies have been suggested, including the use of bran pre-treatments, such as fermentation, and enzymatic or heat treatments, that may enhance the bio-accessibility of phenolics and minimize the negative effects of bran on dough rheology and bread volume [13,14,15]. Alternatively, phenolics can be extracted from wheat bran by ultrasound-assisted technologies and then used as ingredients in pasta and baked products [16,17,18]. Finally, considerable attention has been given to genetic variation among wheat species and cultivars to assess the possibility of improving phenolic acid content in elite wheat germplasm through appropriate breeding programs [19,20,21].

The present review summarizes the chemistry, function, and bioavailability of wheat phenolic acids, and focuses attention on some innovative technologies that can improve the phenolic acid content of final wheat-based products with respect to good taste. Moreover, the review shows the latest literature data on the stability of phenolic acids in pasta and bakery products

## 2. Biosynthesis, Chemistry, and Distribution of Phenolic Acids across the Wheat Grain Tissues

Phenolic acids are plant secondary metabolites belonging to the large family of polyphenols and are generally involved in mechanisms of defense against biotic and abiotic stresses [22,23,24,25,26].

The biosynthesis of phenolic acids is initiated by phenylalanine, the first substrate of the phenyl propanoid pathway, and it proceeds with the synthesis of different phenolic acids and flavonoids [27]. Due to the influence of various biotic and abiotic stimuli on the biosynthetic pathway, phenolic acid content is largely influenced by several factors, such as environmental conditions and agronomic practices [28,29]. Also, the content of phenolic acids depends on genetic components, as well as on genetic per environmental interactions, which result in a large variation among cereal species and cultivars of the same species [20,21,30,31].

Wheat bran phenolic acids can be divided into derivatives of either hydroxycinnamic acid or hydroxybenzoic acid [32]. Hydroxybenzoic acid derivatives include *p*-hydroxybenzoic, vannilic, syringic, and gallic acids. However, the most abundant phenolic acids in wheat are derivatives of hydroxycinnamic acids, specifically ferulic acid, dehydrodimers and dehydrotrimers of ferulic acid, and sinapic and *p*-coumaric acids [20,21,30,33,34,35]. The bran layers contain the majority of phenolic acids that are mostly linked with cell wall structural components through ester bonds [9,36,37].

**Figure 1:**
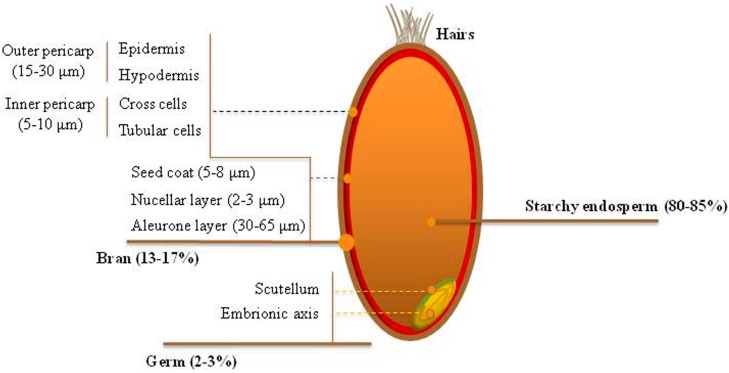
Schematic representation of wheat grain fractions.

Notably, the bran is composed of the aleurone layer, the intermediate layers (e.g., nucellar epidermis and seed coat), the inner pericarp (cross and tube cells), and the outer pericarp [38] (Figure 1).

Table 1 shows the content of principal phenolic acids that are localized in the wheat grain compartments, specifically the bran, the endosperm, the tissues isolated from the outer grain layers, and the scutellum and the embryonic axis. The outer layers of the wheat grain contain a broad array of phenolic acids, including ferulic acid (FA), dehydrodimers (DHD) of ferulic acid, sinapic acid (SA), and *p*-coumaric acid (*p*-CA). As previously noted in maize bran [35], wheat bran also contains a trimer of ferulic acid, namely 4-*O*-8′, 5′-5′′ dehydrotriferulic acid (DHT), which is particularly abundant in the pericarp. The sinapic acid concentration in wheat bran is similar to that of dehydrotriferulic acid, whereas the *p*-coumaric acid content of the outer layers is almost two- and three-fold lower [34].

Also, it was shown that the intermediate layers are mostly composed of arabinoxylan characterized by an extremely low arabinose to xylose ratio, and high amounts of ferulic acid monomers very weakly cross-linked [34]. The aleurone layer differs from the outer pericarp by a lower arabinose to xylose ratio, and a lower content of ferulic acid dimers and trimers. High proportions of ether-linked phenolic acids can be found specifically in the seed coat and tissues in the crease region [35]. It was seen that the recovery of total phenolic acid from the aleurone and nucellar epidermis was similar in mild saponification compared to strong alkali treatment. Conversely, the levels of phenolic acids extracted from the intermediate layer and outer pericarp increased by about two-fold using strong saponification [34].

Phenolic acids have been suggested as biochemical markers of pericarp and aleurone layers [38]. A dehydrotrimer of ferulic acid was found to be mostly concentrated in the outer pericarp of wheat bran, whereas *p*-coumaric acid was mainly present in the aleurone layer [35].

**Table 1:** Contents of principal phenolic acids (mg/g dry matter) of different parts of the wheat grain [34,35]. Abbreviations: FA, ferulic acid; DHD, dehydrodimers of ferulic acid; DHT, dehydrotrimers of ferulic acid; *p*-CA, *p*-coumaric acid; SA, sinapic acid.

Wheat Bran Tissues	FA	DHD	DHT	SA	*p*-CA	Total
Bran	5.26	1.01	0.24	0.25	0.09	6.85
Endosperm	0.10	0.03	0.00	0.01	0.00	0.14
Aleurone	8.17	1.07	0.11	0.44	0.21	10 .00
Intermediate layer	5.92	0.91	0.07	0.08	0.07	7.05
Pericarp	8.18	5.12	1.21	0.01	0.04	14.56
Scutellum	3.48	0.37	0.03	0.01	0.01	3.90
Embryonic axis	0.31	0.09	0.01	0.00	0.00	0.41

Adom *et al.* [9] showed that the bran/germ fraction contributes 83% of the total phenolic content of the whole meal flour. Actually, the total phenolic content of bran/germ fractions is 15- to 18-fold higher than that of respective endosperm fractions and the starchy endosperm contributes only 17% of the total phenolic content [9]. Chiefly, total phenolic content and total antioxidant activity progressively decrease as debranning progresses from the aleurone layer to the internal portions of the kernel [39,40].

## 3. Biological Activity and Bioavailability of Phenolic Acids

Phenolic acids are among the most abundant and ubiquitous metabolites of cereal crops with health-related antioxidant activity [30,32,36]. Indeed, these compounds represent 30% of total phenolics in the Mediterranean diet, thus playing a significant role in both human and animal diets [4,41].

A number of *in vitro* and *in vivo* studies showed that phenolic acids function as free-radical scavengers, reducing agents and quenchers of singlet oxygen formation [42,43]. The antioxidant properties of phenolic acids are mainly attributed to the electron donation and hydrogen atom transfer to free radicals [43]. However, the mechanisms of the antioxidant activity of phenolic acids are also associated with their ability to modify some cellular signaling processes [44]. In particular, hydroxybenzoic acids were shown to activate a number of endogenous anti-oxidant mechanisms, such as the Nrf2 pathway which increases the levels of antioxidant enzymes, thereby decreasing oxidative stress and associated problems such as endothelial dysfunction and inflammation processes [45]. Moreover, some wheat phenolics, such as flavonoids, may act as chelators of trace metals involved in free radical production, but evidence of such activity is not available for phenolic acids [44].

Žilić *et al.* [46] showed that total phenolic and total flavonoid contents were higher in hull-less barley, followed by hull-less oat, rye, durum wheat, and bread wheat. Interestingly, hull-less barley showed the highest antioxidant activity, which was ascribed to a specific subclass of flavonoids. In fact, flavonoids have been suggested to be more effective as antioxidants than Vitamin C, E, and carotenoids [47]. However, to evaluate the link between the antioxidant capacity of cereals and other crop species and the responsible components is not a simple task. The methods used for extraction and identification of phenolic compounds and the evaluation of antioxidant activity may vary considerably, making comparisons between studies and extrapolating general conclusions difficult [48].

When individual phenolic acids were compared for antioxidant activity, ferulic acid showed the highest antioxidant activity compared to caffeic, chlorogenic, cinnamic, gallic, *p*-hydroxybenzoic, protocatechuic, rosmarinic, syringic, *p*-coumaric, and vanillic acids [43]. Ferulic acid possesses three distinctive structural motifs that can contribute to its free radical scavenging capability [49,50]. Rukkumani *et al.* [51] showed that ferulic acid *in vivo* effectively quenches the free radicals and prevents the oxidative stress associated with alcohol and polyunsaturated fatty acids (PUFA)-induced toxicity. Also, the carboxylic acid group, with an adjacent unsaturated C–C double bond, can provide additional attack sites for free radicals, providing some protection against lipid peroxidation [49,50]. In particular, hydroxycinnamates (e.g., ferulic, caffeic, and *p*-coumaric acids) were shown to reduce low-density lipoprotein oxidation, and potentially protect the body from atherosclerosis [52,53].

Production of reactive oxygen and nitrogen species in tissue contributes to the development of various chronic diseases such as cancer, neurodegenerative diseases, and cardiovascular diseases [54,55]. In this context, wheat bran antioxidants might contribute to cellular defense and help to prevent oxidation damage to cellular components. In fact, it has been suggested that the reduction in oxidative damage to cells and cell components may explain the potential of phenolic acids to inhibit cancer and cardiovascular diseases [47]. Nevertheless, the precise effects of phenolic acids are still under debate because of the complexity of studying their biological effects [2,56].

A significant reduction of pro-inflammatory cytokines in humans was also ascribed to phenolic compounds. Effects of ferulic acid on inflammatory messengers in several *in vitro* and *in vivo* models of inflammation were reviewed by Mateo Anson *et al.* [57]. Recent results from an *in vitro* study revealed the potential anti-inflammatory activity of phenolic acids from the whole-meal flour of durum and common wheat [58,59]. In particular, the anti-inflammatory activity of phenolic compounds was shown on human colon cells, suggesting the potential role of these wheat components in intestinal good health [58]. Whole wheat grain consumption was shown to reduce inflammatory processes in a randomized controlled trial on overweight and obese subjects with unhealthy dietary and lifestyle behaviors [60]. Particularly, whole grain consumption for four to eight weeks determined a four-fold increase in serum dihydroferulic acid and a two-fold increase in fecal ferulic acid compared to refined wheat-based foods. Moreover, whole wheat food consumption significantly increased excreted ferulic acid and circulating dihydroferulic acid [60]. *In vivo* experiments showed that polyphenol-rich diets may decrease the risk of chronic diseases by reducing oxidative stress [61].

As a matter of fact, the biological properties of dietary polyphenols depend greatly on their degree of polymerization and bioavailability [62,63,64]. The majority of phenolic acids are insolubly bound by covalent cross-linkages to cell wall polymers, and these bound forms account for approximately 80% of the total phenolic acids [20,30]. Earlier studies suggested a low bioavailability for bound phenolics since they are metabolized in the stomach and small intestine, but only to a less extent [62].

With regard to ferulic acid, an efficient absorption of free forms was found in the intestine [65,66]. The kinetic data of ferulic acid metabolites in urine and plasma after consumption of breakfast cereals revealed that the absorption of ferulic acid from cereals takes place mainly in the small intestine and regards mainly the soluble forms [67]. Further works suggested a different metabolic fate for wheat phenolic over-digestion in humans [68]. In fact, even though the majority of wheat phenolic acids are ester- or ether-linked to polymers of the plant cell wall, intestinal microbes could be able to cleave the bonds, thereby making phenolic acids nutritionally available [69]. Other authors showed that the structural complexity of bound phenolics could explain their health value, since by reaching the colon mostly undigested, they can exert their unique antioxidant and anti-inflammatory activity locally, and contribute to a reduced risk for colorectal cancer [68,69,70].

The role of gut microbiota on the bioavailability and biological activity of high-molecular-weight polyphenols has been recently explored [64,71]. Beside the inter-individual variation in the daily diet intake of polyphenols, differences in the composition of the gut microbiota may occur among different individuals as well, which may result in a diverse grade of bioavailability and bio-efficacy of polyphenols [72,73]. Only a few bacterial species that are involved in such metabolism of phenolics have been identified so far, most of which do not seem to be ubiquitous but reflect the interpersonal differences in the gut microbial community [71,74]. Also, evidence is emerging on how dietary polyphenols can modulate the colonic microbial population composition or activity [63,75].

## 4. Strategies to Increase Phenolic Acid Content and Bioavailability in Wheat-Based Products

The majority of functional components of the wheat grains, namely fibers, vitamins, minerals, and phenolics, are chiefly concentrated in the bran. For this reason, the use of bran in wheat-based products is considered a reasonable way to formulate fortified wheat foods. Indeed, the consumption of whole grain meals is still below dietary recommendations [76,77], which is largely due to the negative effects of bran on dough rheology and the sensory properties of final products [78,79]. It was shown that the addition of bran results in an increase of cooking loss, swelling index, and water absorption in pasta products [10,11,80]. Likewise, a reduced loaf volume, hard crumb, bitter flavor, and dark color were observed in baked products that had been enriched with wheat bran [12,81,82,83]. Several studies showed that the levels of bran addition should not exceed 20% to obtain products with acceptable sensory qualities [18,81,84,85].

Aleurone is considered the most interesting bran fraction to enrich wheat-based products because it contains the highest amount of bioactive compounds and it has the highest antioxidant activity [39,86,87,88]. For this reason, industrial pretreatments and new debranning processes have been developed to obtain the best pearled fractions to be used in bakery and pasta processes in order to maximize the health-promoting effects of bran fiber and phenolics but reduce the negative effects on good taste [89,90,91]. The dietary fiber, beta-glucan, total phenolic and alkylresorcinol content, and the antioxidant activity increased significantly at a replacement level of 10% of pearled wheat fraction while the technological properties were mostly conserved [90].

Recent studies showed that by milling process modulation, it is possible to reduce the particle size of whole wheat flour and wheat bran [92,93]. By using 5% of micronized fractions from the debranning of durum wheat in the dough fermentation of bread wheat flour, it was possible to increase the concentration of free amino acids, total phenols, and dietary fiber and antioxidant activities of dough [92]. The employment of sourdough fermentation made a further improvement of the functional features and of the textural and sensory properties of breads containing bran fractions possible [92].

Pre-treatments of bran, such as fermentation, and enzyme or heat treatments, were shown to enhance the bio-accessibility of nutritional compounds and minimize the negative effects on technological features of end-products [13,14]. By using bran pre-treatments, it was also possible to increase the content of folates, total phenolics, and free ferulic acid [94]. Moreover, yeast fermentation and enzyme addition in whole wheat bread determined an increase in the bioavailability of phenolic compounds [95].

Salmenkallio-Marttila *et al.* [14] studied the effect of bran fermentation on bread-making and found that long fermentation (16 h) with yeast and lactic acid bacteria increased the specific loaf volume and improved the crumb firmness and shelf life of breads with 20% added bran. To further enhance the technological and functional properties of whole wheat foods, phytase can also be added during the bread-making process, resulting in improved specific loaf volume and crumb firmness and in the superior bioavailability of minerals [96,97].

Recent studies showed that the consumption of whole grain products could be hampered by toxicological risks due to the contamination of heavy metals and mycotoxins in the most external layers of the kernel [98]. For this reason, recently, some studies have evaluated the quality of bread enriched with an intermediated pearled wheat fraction of debranning containing the aleurone layers [90,91]. In particular, Blandino *et al.* [90] showed that refined flour can be enriched through the addition of a selected wheat pearled fraction, reducing the risk for deoxynivalenol (DON) contamination. Nevertheless, other studies showed that when a second pearling fraction was used as an added ingredient in bread-making, the content of the DON mycotoxin was reduced by only 32% compared to the refined flour [91]. Thus, accurate checks of the contamination levels of bran and pearling fractions are necessary to make sure that the maximum levels of contamination established by legislation are respected in final products [99].

A genetic approach was also considered to increase the amount of phenolic acids by evaluating the phenolic content and composition among different wheat species and cultivars [100,101]. Genetic variability for phenolic acids was extensively documented in winter and spring bread-wheat genotypes [20,30] and, more recently, in a large number of durum wild and cultivated genotypes [21]. It was shown that phenolic acid variation was only in part due to genetic factors, as a strong influence of environmental factors was observed [19,20]. Indeed, more recent evidence on durum genotypes showed a higher ratio of genotypic variance to total variance, suggesting that it might be realistic to improve phenolic acid content in elite durum wheat germplasm through appropriate breeding programs [21].

## 5. Wheat Bran as a Source of Phenolic Acids for the Food Industry

A number of agri-industrial by-products have been proposed as a source of natural antioxidants [102,103]. Wheat bran, besides being a chief, cheap, and readily available by-product of the cereal industry, is a concentrated source of phenolic compounds. In this context, it can be used to take out edible phenolic extracts for the formulation of various bakery and pasta products.

Among the methods that have been developed for an efficient extraction of phenolic compounds from wheat bran, there is ultrasound-assisted extraction, supercritical fluid extraction, microwave-assisted extraction, and accelerated solvent extraction [17]. These methods have been shown as environmentally friendly because they are able to decrease the solvent consumption, shorten the extraction time, increase the extraction yield, and enhance the quality of extracts [17]. In particular, the ultrasound-assisted technologies make it possible to extract the phenolic compounds from bran without requiring any chemical preliminary hydrolysis. The enhancement in the extraction rate obtained by using ultrasound is mainly attributed to the effects of cavitations produced in the solvent by an ultrasonic wave [16]. The efficiency of methanol, ethanol, and acetone on the extraction of the total phenolic content from wheat bran was evaluated by Wang *et al.* [16]. Results indicated that a significant difference in total phenolic content was observed among the various solvent extracts. Particularly, ethanol recovered the highest content of total phenolics, after methanol and acetone [16]. Moreover, ethanol is a less toxic solvent and can be easily recovered by reduced pressure distillation. Further evidence showed the potential of microwave-assisted solvent extraction to significantly increase the total phenolic compound content in solvents and drastically reduce the extraction time [104].

In a recent study, Delvecchio and Pasqualone [105] found that the enrichment of fresh pasta with phenolic extracts, obtained by preliminary potassium hydroxide (KOH)-induced hydrolysis of wheat bran, negatively interfered both with dough formation and with the sensory properties of the end-product. On the contrary, when aqueous bran extracts were used, the supplemented pasta showed significantly higher antioxidant activity and phenolic content than the control, coupled with good overall sensory properties [18].

Wheat bran is also used to extract an oil that is particularly rich in polyunsaturated fatty acids, vitamin E, carotenoids, and quinones [106,107] to be used as food and a nutraceutical, pharmaceutical, or cosmetic ingredient [107,108]. Due to the high instability of vitamin E and carotenoids and the presence of unsaturated fatty acids that are very susceptible to oxidation, microencapsulation has been proposed as a method to overcome the stability problems typical of bran oils [109,110].

## 6. Stability of Phenolic Acids in Wheat-Based Foods

Several factors may affect the content, composition, and stability of phenolic compounds in wheat-based products as depending on ingredients, industrial, or domestic processing [111,112].

In Table 2, we have summarized the literature studies regarding the modification in free, bound, and total phenolic content in different wheat-based products as affected by baking or cooking processes. The data were adjusted to 100% flour in final products and calculated as percentage difference [111].

In a recent study, the changes in free and bound phenolic acids were estimated in whole grain and lutein-enriched whole grain bread, cookie, and muffin products [111]. All bakery products contained reasonable amounts of phenolic acids. Ferulic acid was the principal phenolic acid both in the free or bound extracts of the six products [111]. Interestingly, it was observed that baking was responsible for an increase in free ferulic acid content in all final products *vs.* whole grain flour (Table 2). The highest increase in free ferulic acid *vs.* flour was observed in whole grain muffins (+99%) and in whole grain lutein-fortified muffins (+95%). On the other hand, a decrease in bound ferulic acid content was observed in bread (38% and 36%) and muffin products (−1% and −6%) *vs.* flour. So far, cookies were the only baking products in which a positive effect of baking on the free and bound ferulic acid contents was observed (Table 2). In general, the increment in free ferulic acid could be due to the release of bound forms from the food matrix during the baking process, which in turn result in a drop-off of bound ferulic acid, especially in bread products [111]. Results showed that the largest effect of lutein-fortification on the increase in free ferulic acid occurred in bread products (Table 2). This might indicate that lutein is more accessible for oxidation and degradation in high-fat bakery products as compared with phenolic acids, whereas in low- or no-fat products, phenolic acids might be more vulnerable to heating and prooxidants [111]. An overall significant reduction of total phenolic content was reported by other authors during the bread-making process [31,90,113], whereas a general increase of total phenolics was observed in muffin products *vs.* flour [114] (Table 2). Although some of the data may look controversial between muffin [114] and bread products [31,90,113], the comparison between different studies is a difficult task due to the various methods used to extract and quantify the phenolic compounds. Moreover, differences in various aspects of product-making processes, such as different ingredients and temperatures, may have contributed to different changes in phenolic contents among the products.

Fares *et al.* [91] evaluated the effect of processing and cooking on the phenolic acid content and composition in fresh pasta products obtained from durum wheat semolina enriched by the addition of debranning fractions of common wheat. The main effect of pasta cooking was a reduction of free and bound phenolic acids, regardless of the kind of flour mixture that had been used (Table 2). Surprisingly, a substantial increase in the contents of bound phenolic acids was found in cooked *vs.* uncooked pasta. The authors suggested that cooking in boiling water was probably responsible for the enhanced extraction of bound phenolics from pasta, especially ferulic acid ester linked to cell walls [91]. Results showed that cooking also affected the antioxidant capacity of pasta by enhancing its antioxidant properties *in vitro*. This could be explained by the Maillard reactions that may take place during the extrusion and drying process of pasta, giving rise to the production of different compounds that exert antioxidant activity [115].

Differently from the results of Fares *et al.* [91], another study regarding commercial spaghetti products showed that the amount of phenolics of spaghetti was negatively affected by the cooking process, with a decrease that ranged from 57% to 68% [116] (Table 2). However, different methods were used in the two studies to extract and quantify the phenolic contents, which might explain such differences.

Overall, various mechanisms have been suggested as responsible for the changes in phenolic acids during baking or cooking processes, including the release of bound phenolics from the food matrix, polymerization and oxidation of phenolics, thermal degradation, depolymerization of high-molecular-weight phenolics such as condensed tannins, and production of Maillard reaction products [117]. Some literature data may look controversial, especially when comparisons are made between studies in which different methods were employed to extract and quantify phenolic acids, or when different ingredients were used in the product-making process. Moreover, in the comparison between studies carried out on bakery and pasta products, it should be considered that temperatures used to develop bakery products are much higher than those used for pasta drying and cooking. Pasta drying is normally used to reduce the moisture content from approximately 30% to about 12%–14% [118]. The parameters that define drying conditions (temperature, humidity, and hair velocity) depend on product shape, dimension, humidity, and load. The most commonly used drying temperatures in traditional processes do not exceed 65 °C, whereas higher temperatures, up to 95 °C, are used in industrial pasta- drying systems. In the studies shown in Table 2, different kinds of pasta products were analyzed fresh [91] or industrially dried [116], which also had a very different shape.

In general, the literature studies showed that bakery and pasta-making processes may result in large modifications of phenolic content and composition, which might have important implications on the bioavailability of some compounds such as ferulic acid [119,120]. Nevertheless, it should be considered that in the human colon, many phenolic acids are also formed from flavonoids and related compounds. For this reason, the biological effects of phenolic acids on human beings are not easily detectable and may not be linked only to the intake of bran or bran-derived food [121,122].

**Table 2:** Changes in free, bound, and total phenolics (TPC) found after the baking of bread, cookies, muffins, and the cooking of pasta products. The literature data were adjusted to 100% flour in final products and calculated as % difference. For each wheat-based product, the ingredients and baking and cooking temperatures and time are shown, as well as the extraction methods used by different authors to determine the phenolic content of different food matrixes. n.a.: data not available.

Product	Ingredients	Baking/Cooking		Extraction Method	Increase/Decrease of Free Phenolic Acids	Increase/Decrease of Bound Phenolic Acids	Increase/Decrease of TPC	Reference
		Temp.	Time					
Unleavened flat bread	Whole grain Einkorn wheat flour, quick yeast, sugar, gluten, water	175 °C	25′	80% methanol; NaOH-hydrolysis	+32% Product *vs.* flour *	−38% Product *vs.* flour	n.a.	[111]
Cookie	Whole grain 1:1 einkorn:corn flour, sugar, salt, sodium bicarbonate, water	175 °C	13′	80% methanol; NaOH-hydrolysis	+35% Product *vs.* flour *	+11% Product *vs.* flour	n.a.	[111]
Muffin	Whole grain 1:1 einkorn:corn flour, sugar, salt, baking powder, margarine, water	175 °C	30′	80% methanol; NaOH-hydrolysis	+99% Product *vs.* flour *	−1% Product *vs.* flour	n.a.	[111]
Fortified unleavened flat bread	Whole grain Einkorn wheat flour, quick yeast, sugar, gluten, water + lutein	175 °C	25′	80% methanol; NaOH-hydrolysis	+17% Product *vs.* flour *	−36% Product *vs.* flour	n.a.	[111]
Fortified cookie	Whole grain 1:1 einkorn:corn flour, sugar, salt, sodium bicarbonate, water + lutein	175 °C	13′	80% methanol; NaOH-hydrolysis	+31% Product *vs.* flour *	+12% Product *vs.* flour	n.a.	[111]
Fortified muffin	Whole grain 1:1 einkorn:corn flour, sugar, salt, baking powder, margarine, water + lutein	175 °C	30′	80% methanol; NaOH-hydrolysis	+95% Product *vs.* flour *	−6% Product *vs.* flour	n.a.	[111]
Bread	Refined wheat flour, salt, brewer yeast, water	215 °C	45′	NaOH-hydrolysis	n.a.	n.a.	−36% Product *vs.* flour	[90]
Enriched bread	Refined wheat flour +10% of second debranning fraction of soft wheat, salt, brewer yeast, water	215 °C	45′	NaOH-hydrolysis	n.a.	n.a.	−35% Product *vs.* flour	[90]
White bread	Commercial wheat flour, salt, sucrose, shortening, dry yeast, water	215 °C	24′	95% ethanol/HCl	n.a.	n.a.	−33% Product *vs.* flour	[113]
Whole wheat bread	Commercial whole wheat flour, salt, sucrose, shortening, dry yeast, water	215 °C	24′	95% ethanol/HCl	n.a.	n.a	−28% Product *vs.* flour	[113]
Purple wheat bran muffins	Purple wheat bran, flour, sugar, baking soda, salt, oil, buttermilk, eggs, molasses	177 °C	20′	100% methanol	n.a.	n.a.	+92% Product *vs.* flour	[114]
Wheat bran Muffins	Flour, sugar, baking soda, salt, oil, buttermilk, eggs, molasses	177 °C	20′	100% methanol	n.a.	n.a.	+90% Product *vs.* flour	[114]
White bread	Commercial wheat flour, salt, sugar, fat, ascorbic acid, fresh yeast, water	220 °C	30′	80% methanol; NaOH-hydrolysis	−20% Product *vs.* flour	17% Product *vs.* flour	n.a.	[31]
Enriched bread	Commercial wheat flour + 6% of second and third debranning fraction of soft wheat, salt, sugar, fat, ascorbic acid, fresh yeast, water	220 °C	30′	80% methanol; NaOH-hydrolysis	−21% Product *vs.* flour	−6% Product *vs.* flour	n.a.	[31]
Enriched bread	Commercial wheat flour + 10% of second and third debranning fraction of soft wheat, salt, sugar, fat, ascorbic acid, fresh, yeast, water	220 °C	30′	80% methanol; NaOH-hydrolysis	−42% Product *vs.* flour	−14% Product *vs.* flour	n.a.	[31]
Enriched pasta	Durum wheat semolina + 6% of first debranning fraction, water	Boiling water	n.a.	80% methanol/1% HCl; NaOH-hydrolysis	−39% cooked pasta *vs.* flour (+0.1% cooked *vs.* uncooked pasta)	-32% cooked pasta *vs.* flour (+26% cooked *vs.* uncooked pasta)	n.a.	[91]
Enriched pasta	Durum wheat semolina + 10% of first debranning fraction, water	Boiling water	n.a.	80% methanol/1% HCl; NaOH-hydrolysis	−36% cooked pasta *vs.* flour; (−30% cooked *vs.* uncooked pasta)	−33% cooked pasta *vs.* flour (+30% cooked *vs.* uncooked pasta)	n.a.	[91]
Enriched pasta	Durum wheat semolina + 6% of second and third debranning fraction, water	Boiling water	n.a.	80% methanol/1% HCl; NaOH-hydrolysis	−37% cooked pasta *vs.* flour (−18% cooked *vs.* uncooked pasta)	−44% cooked pasta *vs.* flour (+27% cooked *vs.* uncooked pasta)	n.a.	[91]
Enriched pasta	Durum wheat semolina + 10% of second and third debranning fraction, water	Boiling water	n.a.	80%methanol/acetic acid; NaOH-hydrolysis	−35% cooked pasta *vs.* flour (−6.8% cooked *vs.* uncooked pasta)	−21% cooked pasta *vs.* flour (+18% cooked *vs.* uncooked pasta)	n.a.	[91]
Regular spaghetti	Durum wheat semolina, water	Boiling water	12′20′′	NaOH-hydrolysis	n.a.	n.a.	−57% cooked *vs.* uncooked pasta	[116]
Spaghetti	Whole durum wheat semolina, water	Boiling water	12′20′′	NaOH-hydrolysis	n.a.	n.a.	−67% cooked *vs.* uncooked pasta	[116]
Fortified spaghetti	Durum wheat semolina, water, inulin	Boiling water	12′20′′	NaOH-hydrolysis	n.a.	n.a.	−68% cooked *vs.* uncooked pasta	[116]

* +, % increase and −, % decrease refer to ferulic acid.

## 7. Conclusions

Phenolic acids vary within different tissues of the wheat grain. A number of studies confirmed the potential of using the intermediate fraction obtained from sequential pearling in bread- and pasta-making as a functional ingredient, as it is a rich source of phenolic acids. Knowledge of the biological activity, bioavailability, biochemistry, and genetics of wheat phenolic acids is quite advanced, but more research is needed. In fact, studies on the impact of wheat phenolic acids on the gut microbiota and their mechanisms of action in humans are scarce. Moreover, as far as DON contamination is concerned, several studies have reported that the outermost kernel layers may have the highest mycotoxin content. The toxicological risks due to mycotoxin contamination could be reduced by using intermediate pearled fractions, but results are still controversial and need further investigation.
